# Individualism and Collectivism as Moderators of Relations between Adverse Childhood Experiences and Adolescent Aggressive Behavior

**DOI:** 10.1007/s10802-025-01296-z

**Published:** 2025-02-27

**Authors:** Hoang-Minh Dang, Trang Le, Cindy Chau, Phuc T. Nguyen, Bahr Weiss

**Affiliations:** 1 Clinical Research Institute for Society, Psychology and Education, VNU University of Education, 144 Xuan Thuy Road, Cau Giay District, Hanoi, Vietnam; 2 Harvard University, Cambridge, MA USA; 3 University of California - Berkeley, Berkeley, CA USA; 4 Vanderbilt University, Nashville, TN USA

**Keywords:** Individualism and collectivism, Adolescent aggression, Adverse childhood experiences, ACE, Vietnam, In-group vs. Out-group

## Abstract

Adverse Childhood Experiences (ACEs) are generally associated with impaired life functioning, including mental health. Prevalent globally, ACEs’ effects vary across cultural settings and groups. The present study assessed horizontal and vertical individualism and collectivism – key cultural dimensions – as moderators of ACEs’ relations to adolescent aggressive behavior. The study was conducted in Vietnam, a Southeast Asian nation with high levels of collectivism but with levels of individualism increasing with globalization. The cross-sectional study included 644 high-school students (mean age = 16.6 years; 54% female). Self-report measures assessed ACEs, adolescent aggressive behavior, and individualism and collectivism. Given collectivism’s focus on supporting one’s social communities, it was hypothesized that collectivism would serve as a protective factor for statistical effects of ACEs on aggression. All four moderator analyses involving collectivism supported this hypothesis; i.e., at high levels of collectivism, relations between ACEs and aggression were significantly smaller than at low levels of collectivism. Statistical effects were largest for vertical (endorsing hierarchical power structures) collectivism. In the one significant individualism moderator analysis, individualism served as a risk factor for (i.e., increased) statistical effects of ACEs on proactive aggression. Results highlight the importance of the horizontal-vertical sub-dimensions, as they may provide for more precise theoretical explanatory and intervention models. For instance, concern for the power hierarchy’s reactions (vertical collectivism) to one’s aggression vs. concern for harm to one’s groups (horizontal collectivism) by one’s aggression have different implications for prevention and treatment that may be useful for maximizing interventions’ effectiveness.

**Supplementary Information:**

The online version contains supplementary material available at 10.1007/s10802-025-01296-z.

## Introduction

Adverse childhood experiences (ACEs) are traumatic or stressful events occurring during childhood (0 to 17 years of age) that typically involve emotional, physical, or sexual abuse, serious family dysfunction, and/or exposure to community or collective violence (Metzler et al., 2017). ACEs are prevalent across a wide range of international and cultural settings (Cluver et al., 2015; Le et al., 2022). ACEs have been linked to a variety of negative health-related outcomes, including health risk behaviors such as smoking or a sedentary life style (Ford et al., 2011; Hillis et al., 2001) and chronic physical diseases (Gilbert et al., 2015; Rehkopf et al., 2016). They also are associated with risk for various forms of mental health conditions including depression and anxiety, PTSD and behavioral conduct problems (Reijntjes et al., 2011; Strathearn et al., 2020).

ACEs’ relations to various health-related outcomes have been found to vary across ethnic groups and as a function of cultural factors (Dang et al., 2022). Elkins et al. (2019) for instance found that the risk for PTSD associated with ACEs was significantly larger for Euro-American adolescents than for Hispanic American and African American adolescents. Research focused on other areas of mental health functioning (e.g., substance abuse; Johnson et al., 2023) has found similar ethnic and racial differences. Such results suggest that cultural factors may moderate – either exacerbate or ameliorate – relations between ACEs and health-related functioning (Korbin, 2002; Vaughn et al., 2015). Culturally-related moderators of relations between ACEs and health functioning have been identified, including the extent to which one’s personality is congruent with broader societal values (Caldwell-Harris & Ayçiçegi, 2006), and cultural variations in social support networks (Brockie et al., 2018). Upenieks et al. (2024) for instance found that higher levels religious involvement and use of “negative religious coping techniques” (e.g., viewing God as harsh and judgmental) were associated with larger relations between emotional neglect and abuse, and depressive symptoms. In regard to aggressive behavior, a small number of studies have identified cultural moderators of ACEs’ effects but such moderators have not been studied in detail. In a study involving Asian-American adolescents, Ngo and Le (2007) reported a significant moderator effect for collectivism on relations between physical abuse and violent adolescent behavior, but details of the moderator effects were not reported. Understanding such processes is critical, as they potentially will help to delineate more precise theoretical explanatory models for health-related effects of ACEs.

### Individualism and Collectivism

Individualism and collectivism are among the most fundamental dimensions used to describe cultural variations, representing contrasting but not bipolar orientations regarding relationships between individuals, and the groups, society, and structures within which the individuals reside (Triandis, 1995). “Collectivism” refers in part to the extent to which individuals and societies value and behaviorally focus on cohesive and interdependent social groupings emphasizing the needs and values of the group (Triandis & Gelfand, 2012). Central aspects of collectivism include (a) one’s identity being primarily derived from one’s group membership(s), (b) with supporting others in one’s groups a major focus, (c) group norms and varying styles of enforcement of group norms (e.g., egalitarian; authoritarian) a key influence on one’s behavior (Oyserman et al., 2002; Singelis et al., 1995). Within collectivism, the social groups of one’s focus typically can range from one’s nuclear family, to one’s extended family, to friends and local communities, etc., up to broader geographic groups including the national level, with different emphasis across persons and cultures on the relative importance of the groups (Triandis & Gelfand, 2012). The groups to which one has a sense of connection have been referred to as “in-group”, in comparison to “out-group” which refers those groups to which a person does not have a sense of connection (Triandis, 2001); responses and commitment to in-group vs. out-group persons can vary significantly (Triandis, 2001). “Individualism” in contrast refers in part to viewing oneself as an autonomous agent distinct from any group and from others in such groups, with a focus on one’s own attributes and accomplishments rather than on group affiliation. Central aspects of individualism include (a) a sense of identity primarily derived from one’s own specific characteristics, with (b) high value placed on independence and on self-reliance, with a focus on (c) one’s rights as an individual, and on (d) one’s goals and personal achievements (Bergmüller, 2013; Hofstede, 1991; Hui & Triandis, 1986).

Beyond these basic elements, individualism and collectivism are seen as involving two critical sub-dimensions, the horizontal dimension and the vertical dimension (Shavitt et al., 2010). The horizontal dimension involves the value and emphasis placed on equality among persons within one’s world, whereas the vertical dimension involves one’s acceptance of and styles of interaction with the power hierarchies within one’s world. Similar to individualism and collectivism, the horizontal and vertical dimensions are seen as contrasting but not bipolar orientations. In brief, horizontal individualism involves seeing oneself as unique from others, but valuing and believing in the general equality of persons within the world. Vertical individualism also involves seeing oneself as unique and independent, but believing that some persons (potentially including oneself) are of more value, and deserve or have earned higher status and power within this society of individuals. Horizontal collectivism involves the perspective that one is fundamentally defined and supported by the in-groups (family, peers, work, government, etc.) within which one resides, with valuing, believing in, and striving for equality of the persons within these groups. Vertical collectivism also involves the perspective that one is defined by the groups within which one resides, but with acceptance and respect for differences in status, treatment (by the hierarchy), and power within and between these groups (Singelis et al., 1995).

Individualism and collectivism have been found to be significantly related to youth conduct problems. For instance, (T. N. Le & Stockdale, 2005) found in a sample of Asian-American youth that individualism had a significant positive relation to delinquency, with higher levels of individualism associated with higher levels of delinquency. In contrast, collectivism showed a significant negative relation with delinquency, with higher levels of collectivism associated with lower levels of delinquency.

Theoretical frameworks (e.g., resilience) can be of significant value in science for understanding and guiding research, including research focused on influence of cultural factors, as above. A key value of theoretical frameworks is that they can organize hypothesized processes into coherent models that suggest additional potential components and hypotheses (Bingham et al., 2024). One resilience-related model compatible with the current study’s setting (a country with high levels of vertical collectivism) and focus (aggressive behavior) is Social Control Theory (Costello, 2017; Hirschi, 1969). In brief, Social Control Theory (SCT) posits that having strong, positive bonds to other persons, social institutions, and authority figures (i.e., having high levels of vertical collectivism) promotes adherence to social rules, including resilience in the face of stress (e.g., in the present instance, ACEs). This in turn reduces deviant, norm-violating behavior such as aggression. Key SCT processes are (a) belief in the meaningfulness and validity of societal norms and power structures, which motivates development of self-regulation skills; (b) attachment and emotional connections to parents or caregivers or other central adults, creating a desire to avoid upsetting them, motivating development of social skills; and (c) commitment and investment in socially valued activities (e.g., education), which discourages risk-taking that could disrupt related goals, and provides role models and learning for appropriate behavior, including coping (Costello, 2017; Hirschi, 1969).

Components of individualism and collectivism within the context of the Social Control Theory suggest possible mechanisms for such effects. In regard to individualism, persons who see themselves as independent entities and are primarily focused on their own success and well-being (i.e., persons high on individualism) may be more willing to break rules and be more aggressive in seeking their own advancement and achievement, since these are of primary concern. In contrast, person’s whose sense of identity comes from their social groups and who are concerned about social welfare and social acceptance may be less likely to break rules or be aggressive due to their concern for group and societal welfare potentially harmed by such delinquency, and due to concerns of negative group reactions (e.g., the family) to their delinquency or embarrassment caused to the key in-groups such as the family (“family shame”) (Le & Stockdale, 2005; Park et al., 2021).

Individualism and collectivism thus potentially represent important moderators of relations between ACEs and life functioning, including conduct problems. Research in this area is limited (Ngo & Le, 2007), however, and to the best of our knowledge to date no published studies have assessed moderation of ACEs’ effects by horizontal and vertical individualism and collectivism. Use of the horizontal and vertical sub-dimensions is critical, as these sub-dimensions may provide more precise empirical and theoretical understanding of processes underlying moderation. For instance, adolescent conduct problems often involve conflict with authority (Butcher & Kendall, 2018). Thus, links to attitudes towards and relations with authority and power structures that the vertical dimension involves may provide increased detail regarding relations between individualism and collectivism, and adolescent conduct problems, which could potentially provide for more culturally-congruent interventions, and increased effectiveness.

### Current Study

The goal of the current study was to assess moderator effects of horizontal and vertical individualism and collectivism on relations between ACEs and aggressive behavior, among adolescents in the Southeast Asian nation of Vietnam. Historically, Vietnam has been and remains a strongly collectivistic country (Hofstede, 2024), with a pronounced and patriarchal focus on the family. The culture’s hierarchical aspects can result in strong competition among members of the culture, with for instance a child’s academic success (or failure) seen not just as individual achievement (or failure) but also as a competitive reflection of the family’s social standing (London, 2015). Similar to many other non-Western nations, as the country’s economy and society have globalized, levels of individualism have begun to increase, providing for a broader range of cultural variation (Ho et al., 2021).

Adolescent aggressive behavior was the primary study outcome. Although ACEs are associated with a wide range of mental health problems, the study focused on aggression given its importance from a public health perspective. Aggressive behavior not only impacts the individual exhibiting the behavior (e.g., with increased risk for interaction with the criminal justice system, or for academic failure; Reavis et al., 2013; Kashif et al., 2022) but also perpetuates a cycle of violence onto others. The present study focused on two types of aggressive behavior, proactive aggression and reactive aggression as these two forms of aggression have underlying causal processes potentially influenced by individualism and collectivism. Proactive aggression involves aggressive behavior that is premeditated, goal-oriented, and instrumental (i.e., influenced by its positive, or negative, consequences) (Dodge & Coie, 1987). Key factors in development of proactive aggression are observational learning (from peers, parents, etc.) regarding the benefits and reinforcement versus the costs and punishment of proactive aggression as well as its social acceptability (i.e., norms) (Romero-Martínez et al., 2022). Reactive aggression involves emotional and behavioral over-reactions to perceived threats or frustrations, and is generally unplanned and impulsive (Dodge, 1991). Key factors in development of reactive aggression are high levels of emotional reactivity and a cognitive bias towards perceiving threats in ambiguous situations (Romero-Martínez et al., 2022). The present study focused on adolescents, given ACEs by definition occur during childhood and adolescence, and because onset of aggression and related behaviors often occurs early in life (Malti & Rubin, 2018).

#### Hypotheses

Study hypotheses for main effects were that (1) collectivism would show a negative relation with adolescent aggression (i.e., the higher the collectivism, the lower the aggression) and (2) individualism would show a positive relation with aggression (i.e., the higher the individualism, the higher the aggression). Hypotheses for moderator effects were that (3) collectivism would serve as a protective factor for the statistical effects of ACEs on adolescent aggression (i.e., the higher the collectivism, the smaller the relation between ACEs and aggression). This hypothesis was based on the fact that collectivism is defined by concern for and connections to one’s social groups, thus potentially reducing behaviors harmful to others or resulting in negative reactions from others. Hypothesis (4) was that the strongest protective moderator effect within collectivism would be vertical collectivism. The “vertical” sub-dimension of collectivism involves acceptance and respect for differences in power hierarchies within one’s community. This hypothesis was based on the fact that perpetrators of ACEs are often individuals higher in social hierarchies (Suprina & Chang, 2005), and that individuals with higher levels of vertical collectivism thus may be less reactive to ACEs because of their acceptance of the power differentials that ACEs fundamentally represent (Suprina & Chang, 2005). In regard to individualism, it was hypothesized (5) that individualism would serve as a risk factor for the statistical effects of ACEs on adolescent aggression, with higher levels of individualism associated with larger relations between ACEs and adolescent aggression. This was hypothesized because for individuals high in individualism, ACEs could provide models of the value of aggression (affecting proactive aggression), and potentially result in increased anger and emotional reactivity (affecting reactive aggression) for the recipient of the ACEs because of perceptions of their individual rights being violated. Finally, it was hypothesized (6) that individualism would be a less powerful moderator than collectivism with smaller, and / or a fewer of the moderator effects significant, because individualism is less normative in Vietnam compared to collectivism, potentially resulting in less social support for these reactions.

## Methods

### Participants and Sampling Frame

The goal of the sampling frame was to provide a sample representing a range of socio-economic conditions in Vietnam to enhance generalizability. Hanoi and Hung Yen provinces were purposively selected to include (respectively) (a) more highly developed / Westernized urban areas, as well as (b) lesser developed / less Westernized rural areas. Within each province, one high school in a relatively urban area and one in a relatively rural area were selected randomly. In Vietnam, high-school consists of grades 10, 11, and 12. The present study excluded grade 12 as students in this grade are focused on preparing for college entrance exams and generally do not have time for non-academic activities. In each school, stratified random sampling (by grade) was used to select approximately 100 students from grade 10 and from grade 11. Depending on class size, two or three classes were selected randomly within grade within school. A total of 800 students from the four schools were invited to participate in the study. Parent consent and adolescent assent were obtained from 723, for an 89% recruitment rate. Participants with an invalid survey (e.g., fixed pattern responses; > 10% missing items) were not included in analyses, with 644 participants (89%) considered to have valid data.

### Measures

The survey included four components: (a) a demographic measure assessing basic participant information; (b) proactive and reactive aggressive behavior (dependent variables); (c) adverse childhood experiences (independent variables); and (d) individualism and collectivism (moderator variables). Measures for which there was not an extant Vietnamese version were culturally adapted, translated, and back translated by a bilingual team of psychologists in Vietnam and the U.S. using standard procedures to maintain measures’ semantic and conceptual content (Byrne, 2016; Hambleton, 2005). The team is highly experienced in measure adaptation and translation, being the official translator and distributor in Vietnam for a number of youth assessment instruments (e.g., WISC-IV, Dang et al., 2011; Child Behavior Checklist, Dang et al., 2017). Internal consistency was assessed using the omega statistic (Dunn et al., 2013), with a minimum acceptable internal consistency value of 0.50 (George & Mallery, 2021; Gliem & Gliem, 2003). Because internal consistency estimates are significantly biased when a scale has a small number of items (Thompson et al., 2010; Vaske et al., 2017), internal consistency was not computed for scales with less than five items. The Adverse Childhood Experiences scales were not assessed for internal consistency as internal consistency is generally not seen as meaningful for scales assessing life events (Harkness & Monroe, 2016).

#### Adolescent Aggressive Behavior

The Peer Conflict Scale (PCS) is a 40-item self-report scale measuring adolescent proactive and reactive aggression (Marsee et al., 2011). Items are rated on a four-point Likert scale, from 0 (Not at all true) to 3 (Definitely true). Proactive Aggression includes items such as “*I start fights to get what I want”;* Reactive Aggression includes items such as “*I threaten others when they do something wrong to me*”. As the wording of these examples suggests, items target behaviors potentially directed towards but not restricted to peers. The PCS was adapted and translated using the procedures described above. Omega internal consistency was ω = 0.79 for Proactive Aggression and ω = 0.82 for Reactive Aggression.

#### Adverse Childhood Experiences

The Adverse Childhood Experiences – International Questionnaire (ACE-IQ; (World Health Organization, 2018) assessed adverse childhood experiences. The ACE-IQ consists of (a) 29 items assessing (b) 13 types of ACEs, in (c) 7 domains: (1) Neglect (emotional; physical), (2) Abuse (emotional; physical; sexual), (3) Family Dysfunction (e.g., family member who has been sent to prison), (4) Family Violence (witnessing domestic violence), (5) Peer Violence (being bullied, being in repeated physical fights), (6) Community Violence (e.g., witnessing people outside the home being attacked) and (7) Collective Violence (exposure to war or gang violence). Participants answer items regarding their experiences up to age 18. The ACE-IQ Likert scale scoring (WHO, 2018) was used for this study. Likert scales differ across different ACE-IQ domains. For instance, Emotional Neglect Item #1 (parent understanding of their child’s emotional life), is scored from 1 (Always) to 5 (Never). For Physical Neglect Item #2 (a parent so intoxicated they cannot take care of their child), the scale ranges from 1 (Many times) to 4 (Never). As discussed in statistical analyses section below, the eight ACE domains were combined into a single variable, by converting all items from their original scoring to a 0 (no problem) to 3 (worst) scale, and taking the item sum (WHO, 2018). The ACE-IQ has been culturally adapted and used in a wide range of countries (WHO, 2018), including Vietnam (Tran et al., 2015). In the current study, the Tran et al. (2015) version of the ACE-IQ was used but added back the emotional neglect and collective violence items not included in Tran et al. (2015), following the procedures described previously.

#### Individualism and Collectivism

The Individualism and Collectivism Scale (INDCOL; Triandis & Gelfand, 1998) is a 16-item questionnaire that measures horizontal and vertical individualism and collectivism. Items are rated on a 9-point Likert scale, ranging from 1 = *“totally disagree”* to 9 = *“totally agree”*. The Individualism scale and the Collectivism scale each have two subscales, Horizontal and Vertical. The Horizontal Individualism subscale includes items such as “I’d rather depend on myself than on others”. The Vertical Individualism subscale includes items such as “Competition is the law of nature”. Horizontal Collectivism involves items such as “If a fellow student gets a prize, I would feel proud”. Vertical Collectivism includes items such as “It is important to me that I respect the decisions made by my groups”. The INDCOL was adapted and translated using the procedures described above. Because the four subscales each had four items, internal consistency was not assessed. To assess the construct validity of the Vietnamese version of the INDCOL, a series of construct validity analyses were conducted. *Supplemental Materials #1 – Construct Validity of the INDCOL Scale /* Supplemental Table 1 reports results of these analyses; all three construct validity hypotheses were confirmed.

### Procedures

An introduction letter was first sent to each school’s principal; all four principals provided permission for their school’s participation. Within 10th and 11th grades, classes were randomly selected. For student recruitment, in each participating class a research assistant introduced the study, answered any questions about the study, and provided interested students with a study description and consent form for the parents. Only students who returned a signed parental consent form who also provided their own signed assent participated in the study. Data collection was conducted in a group meeting in a classroom during a free period, with research assistants providing an overview of the questionnaire, response categories, etc. Participants then completed the questionnaire, which took about thirty minutes. The survey was anonymous (i.e., students’ names were not on the survey, and consent and assent forms were not linked to the survey), and teachers were not present in the classroom. Each participating classroom was given one million Vietnamese dong (approximately US $50) for use by the teacher for classroom activities.

The study was reviewed and approved by the Institutional Review Board of the VNU University of Education-Hanoi, Vietnam. To ensure full confidentiality and increase data validity, participants’ responses were not linked to their name or other identifying information, and there was no linkage between questionnaires and consent and assent forms. Participants were informed that anything on questionnaires would not and could not be linked to them. Participants were also informed that if a participant reported some form of abuse to the research assistant, following Vietnamese regulations it would be reported to child protective services. During data collection, no participant mentioned or reported any reportable behavior (i.e., abuse).

### Statistical Analyses

Missing data was treated as missing (i.e., not estimated). Inferential analyses were conducted using a general linear models (GLM) framework within SAS (9.4) Proc GLM. To interpret significant interactions, the SAS Proc GLM Estimate function was used to compute the slope, standard error, and statistical significance for the relation between the independent variable and dependent variable at -1 SD and + 1 SD from the mean of the moderator. For inferential analyses involving ACEs, we first tested whether moderator effects of the INDCOL varied significantly as a function of ACE Domain, using a repeated measures framework. They did not (see Preliminary Analyses, below), so the eight ACE domains were combined into a single variable.

## Results

### Descriptive Statistics

The mean age of the participants was 16.58 years (SD = 0.50), with 43% in the 10th grade, and 54% female. Table 1 reports bivariate correlations among the primary study variables.

**Table 1:** Bivariate correlations

	Proactive Aggress	Reactive Aggress	ACE Total	Collect Horiz	Collect Vert	Indiv Horiz	Indiv Vert
Proactive Aggression	1.00						
Reactive Aggression	0.65 ****	1.00					
ACE Total Score	0.33 ****	0.38 ****	1.00				
Collectivism - Horizontal	− 0.20 ****	− 0.16 ****	− 0.04	1.00			
Collectivism– Vertical	− 0.23 ****	− 0.11 **	− 0.11 **	0.41 ****	1.00		
Individualism - Horizontal	0.04	0.09 *	0.11 **	0.11 **	0.22 ****	1.00	
Individualism - Vertical	0.18 ****	0.20 ****	0.07	0.01	0.13 **	0.34 ****	1.00

Notes: Proactive Aggress = Proactive Aggression; Reactive Aggress = Reactive Aggression; ACE Total = ACE Total Score; Collect Horiz = Collectivism – Horizontal; Collect Vert = Collectivism – Vertical; Indiv Horiz = Individualism Horizontal; Indiv Vert = Individualism Vertical. *p* <.05 ^*^; *p* <.01 ^**^; *p* <.001 ^***^; *p* <.0001 ^****^

### Preliminary Analyses

To determine whether individual ACE domains should be analyzed separately or combined into a total score, we tested whether moderator effects of IC/HV differed as a function of ACE Domain. Proactive Aggression and Reactive Aggression were dependent variables in separate models. ACE Domain was a repeated measure factor (df = 7), with each of the four IC/HV variables as moderator in separate models, for a total of eight models. The effect of interest was the ACE by IC/HV by ACE Domain interaction term. *Supplemental Materials #2 -* Supplemental Table 2 reports these results. For Vertical Collectivism, the ACE Domain X Vertical Collectivism interaction on Proactive Aggression was significant, F(7,587) = 2.50, *p* <.05, ω^2^ = 0.02. The other seven interaction tests were non-significant. Because the number significant effects (1 of 8) for ACE Domain was not greater than expected under the null hypothesis (χ2[1] = 0.28, ns), study analyses used the ACE Total Score variable, combined across ACE Domain.

### Main Effect Relations with Adolescent Aggression

Main effect relations between ACEs and adolescent aggression were conducted to provide a baseline for moderator effects. Main effects relations between ACE Total Score, and (a) proactive aggression and (b) reactive aggression were assessed in separate models. A general linear model analysis with standardized parameter estimates was used. Table 2 reports results of these analyses. Both dependent variables showed the same pattern, with higher levels of ACEs associated with higher levels of aggressive behavior, with similar effect sizes for proactive aggression (β = 0.33) and reactive aggression (β = 0.38).

**Table 2:** Main effects of ACE, and collectivism and individualism on peer aggression

Independent variable	Dependent variable			
	Proactive Aggression		Reactive Aggression	
ACE Total Score	F(1,642) = 79.04^****^	β = 0.33	F(1,642) = 107.44^****^	β = 0.38
Horizontal Collectivism	F(1,642) = 28.15^****^	β = − 0.20	F(1,642) = 16.16^****^	β = − 0.16
Vertical Collectivism	F(1,642) = 35.09^****^	β = − 0.23	F(1,642) = 7.66^**^	β = − 0.11
Horizontal Individualism	F(1,642) = 0.96	β = 0.04	F(1,642) = 5.28^*^	β = 0.09
Vertical Individualism	F(1,642) = 20.76^****^	β = 0.18	F(1,642) = 25.46^****^	β = 0.20

Notes: ^*^= *p* <.05, ^**^= *p* <.01, ^***^= *p* <.001, ^****^= *p* <.0001 for relation between the independent and dependent variable

#### Individualism and Collectivism

We next tested main effects between Horizontal and Vertical Individualism, and Horizontal and Vertical Collectivism, with Proactive Aggression and Reactive Aggression as the dependent variables, using the same statistical models. Seven of eight relations were significant. For all of its significant effects, individualism had a positive beta with aggression (i.e., the higher the individualism, the higher the aggression) whereas in contrast for all of its significant effects, collectivism had a negative beta with aggression (Table 2).

### Moderator Effects

Main study analyses focused on moderator effects of horizontal and vertical individualism and collectivism (IC/HV) on relations between ACEs and adolescent aggression. To determine whether Location (urban vs. rural) should be included in analyses, we tested whether the moderator effects of IC/HV differed as a function of Location, with the ACE x IC/HV x Location moderator the effect of interest, with all component lower-order terms included in the models. The three-way interaction term was significant for one of the eight models (see *Supplemental Materials #3–* Supplemental Table 3). Because the number significant effects (1 out of 8) for Location as a moderator of ACE by IC/HV effects was not greater than expected under the null hypothesis (χ2[1] = 0.28, ns), the primary analyses did not include Location.

Thus, in the primary analyses the independent variable was ACE Total Score, moderators were the four INDCOL IC/HV subscales in separate models, with dependent variables proactive and reactive aggression, totaling eight analyses (see Table 3). Figures 1 and 2 (see Supplemental Materials #4 – Figures) provide graphic representation of the results. All four moderator analyses involving collectivism were significant, with the same pattern: At low levels of collectivism (including both horizontal and vertical collectivism), relations between ACEs and proactive and reactive aggression were significant, and larger than at high levels of collectivism; i.e., collectivism served as a protective factor for the statistical effects of ACEs on aggression. The largest moderation effect (ω^2^ = 0.063) was for vertical collectivism with proactive aggression as the dependent variable. One of four analyses involving individualism was significant, with vertical individualism serving as a moderator of the statistical effects of ACEs on reactive aggression. In contrast to the moderator effects of collectivism, high levels of individualism were associated with a larger relation between ACEs and reactive aggression; i.e., individualism served as a risk factor for the statistical effects of ACEs on reactive aggression (see Table 3).

**Table 3:** Moderator effects of Horizontal and Vertical Collectivism and Individualism on relation between ACE and adolescent aggression

Moderator	Dependent Variable	Moderator effect		Interaction breakdown^1^	
		F(1,640)	ω^2^	β @ -1 SD	β @ +1 SD
Horizontal Collectivism	Proactive Agg	12.53***	0.018	**0.43**	**0.19**
	Reactive Agg	6.58*	0.009	**0.45**	**0.27**
Vertical Collectivism	Proactive Agg	44.25****	0.063	**0.46**	0.08
	Reactive Agg	12.25***	0.017	**0.45**	**0.25**
Horizontal Individualism	Proactive Agg	0.01			
	Reactive Agg	0.45			
Vertical Individualism	Proactive Agg	0.00			
	Reactive Agg	4.74*	0.006	**0.29**	**0.45**

Notes: **ω**^**2**^ = partial omega-squared. ^*^= *p* <.05, ^**^= *p* <.01, ^***^= *p* <.001, ^****^= *p* <.0001. DV = Dependent variable. The independent variable in these models is ACE Total Score.^1^ = β is standardized beta for relation between the particular IV and DV, at +/-1 SD of the Moderator from the mean. **β** in **bold** are significantly different from zero

Finally, analyses were conducted to assess whether the significant moderator effects of collectivism differed as a function of the horizontal versus vertical sub-dimensions. In the first analysis, proactive aggression was the dependent variable, with the independent variables ACE, horizontal collectivism, vertical collectivism, and the two interaction terms ACE x horizontal collectivism, and ACE x vertical collectivism. The GLM model tested whether the beta for ACE x horizontal collectivism versus ACE x vertical collectivism differed significantly. The test was significant, F(1,638) = 8.95, *p* <.005, indicating that the moderator effects of horizontal collectivism versus vertical collectivism on relations between ACE and proactive aggression differed significantly, with the moderator effect size for vertical collectivism larger than for horizontal collectivism (see Table 3). The same analysis was conducted with reactive aggression as the dependent variable; moderator effects of horizontal versus vertical collectivism did not differ significantly, F(1,638) = 0.67, ns. These analyses were not conducted for individualism, because only one of the four moderator analyses was significant.

## Discussion

This study is, to the best of our knowledge, the first published assessment of statistical moderation by horizontal and vertical individualism and collectivism of relations between ACEs and youth functioning. Results indicated that collectivism served as a protective factor, significantly reducing relations between ACEs and adolescent aggression, with vertical collectivism the strongest protective factor. This is important since understanding such processes can be a critical step in development of supportive interventions, by identifying most central risk and protective factors to be targeted by interventions (Jepson et al., 2022). Main effects were first analyzed to provide description of base processes underlying moderation (Hayes, 2013). As hypothesized, main effects of ACEs on proactive and reactive aggression were significant, with higher ACEs associated with higher adolescent aggression (see Table 2). Proactive aggression involves aggressive behavior that is premeditated, goal-oriented, and instrumental (Dodge & Coie, 1987). Key factors in development of proactive aggression are observational learning regarding (a) its social acceptability or lack thereof (i.e., norms), and (b) the potential benefits and potential costs (e.g., punishments) of proactive aggression (Romero-Martínez et al., 2022). The positive relation between the ACEs and proactive aggression thus may reflect at least in part an adolescent modeling the violence and / or disregard for others they experience and observe as part of the ACEs, as they observe the benefits (e.g., social status; control of one’s environment) potentially gained from violence, and its social acceptability (i.e., norms) within their social context (Shu & Luo, 2021; Werner & Nixon, 2005). Reactive aggression in contrast involves unplanned emotional and impulsive behavioral over-reactions to perceived threats or provocations (Dodge, 1991). Key factors in development of reactive aggression are (a) high levels of emotional reactivity and (b) a cognitive bias towards perceiving threats or challenges in ambiguous situations (Romero-Martínez et al., 2022). Thus, the positive relation between ACEs and reactive aggression may reflect at least in part the adolescent experiencing fear and anger from being subjected to ACEs, resulting in increased emotional reactivity, a protective tendency to view ambiguous situations as threats, and consequent increased reactive aggression.

ACEs can impact on neurobiological development, affecting the amygdala, hippocampus and other brain structures (Herzog & Schmahl, 2018), which may impact on a person’s functioning in a range of ways. For instance, such neurobiological effects can result in impairment in executive functioning, coping capacity, and emotion regulation (Cabrera et al., 2020), and thus underlie behavioral and cognitive processes linking ACEs and mental health functioning. Understanding such ACE-related neurobiological processes is in its early stages but represents an important area for understanding processes underlying moderator effects (Cabrera et al., 2020).

Seven of eight main effects between individualism and collectivism with proactive and reactive aggression were significant (see Table 2). The three significant relations involving individualism (proactive aggression, and vertical individualism; reactive aggression, and horizontal and vertical individualism) all showed the same pattern, with higher individualism associated with higher adolescent aggression. The pattern of significant / non-significant relations may reflect in part (a) individuals high on vertical individualism choosing to use proactive aggression to further their achievement goals and self-interest, as they view competition (including aggression) and hierarchy as an inherent part of reality; (b) individuals high on horizontal individualism not using proactive aggression more than other persons because their belief in equality among people that is part of horizontal individualism balances out potential benefits seen for aggression furthering one’s self-interest; and (c) individuals high on horizontal and vertical individualism reacting more strongly with anger and fear to threats to their independence and success in their lives, resulting in increased reactive aggression.

All four collectivism main effects were significant, and showed the same pattern, with higher collectivism associated with lower aggression. The fact that relations were in the same direction and of similar magnitude for horizontal and vertical collectivism suggests that the horizontal and vertical sub-dimensions did not play a major role in these main effects. This in turn suggests that it may be the central focus of collectivism, concern for the welfare of the group, and concern about negative social consequences of violating one’s normative position in one’s groups that underlies these relations. High levels of these factors (high collectivism) may result in (a) the costs of aggression (harm to the group; rejection by the group) over-riding benefits one might observe from aggression, leading to reduced proactive aggression, and (b) increase adolescents’ trust and emotional stability, leading to reduced reactive aggression (Triandis & Gelfand, 2012).

### Moderator Effects

The study’s primary focus was on the moderating effects of individualism and collectivism on relations between ACEs and adolescent aggression. As hypothesized, all four interactions with collectivism were significant and showed the same pattern, with higher levels of collectivism associated with a reduced or non-significant relation between ACEs and adolescent aggression; i.e., high collectivism was a protective factor for relations between ACEs and adolescent aggression (Table 3). Vertical collectivism showed the largest of these moderator effects, on proactive aggression. The primary focus of vertical collectivism involves respect for and acceptance of the power hierarchy necessary for the welfare of and acceptance by one’s group(s), with one’s family a central group in which one resides (Triandis & Gelfand, 1998). In regard to proactive aggression as the dependent variable, this suggests that it may be awareness that although aggression might benefit oneself (the adolescent), it also could cause harm to or rejection by or to one’s groups (e.g., “family shame”) as a consequence of the power hierarchy’s reaction to the adolescent’s proactive aggression.

The horizontal versus vertical collectivism moderator effects on reactive aggression did not significantly differ, which suggests that it is the general focus of collectivism, a sense of connection and identity to one’s social groups that underlies these moderator effects. It may be that this sense of connection to the group and one’s sense of identity coming from the group results in less emotional reactivity in response to ACEs (“stoicism”), and consequently less reactive aggression.

Only one of four individualism moderator relations was significant, with its ω^2^ statistic smaller than the ω^2^ for the four collectivism moderator effects; i.e., although beliefs in individualism were associated with increased levels of aggression as main effects, they showed relatively little influence on relations between ACEs and aggression. It is possible this is a result of individualism being linked to increased aggression, as seen in the main effects, more via general attitudes towards the self (e.g., the importance of furthering one’s self-interest) rather than via reaction to specific events such as ACEs. It is also possible that individualism’s main effects on aggression do involve response to specific events but ACEs represent a different type of event vis-à-vis aggression, perhaps because ACEs often involve the self (e.g., being bullied) versus for instance events not directly involving the self (e.g., observing a peer being bullied).

### Implications

Social Control Theory structure (as discussed above) highlights the importance of multi-component interventions focused on different elements underlying social control. One related implication of the present findings is that making adolescents aware of potential consequences of both their aggressive behavior but also of alternative pro-social, non-aggressive behavior – interventions frequently used to reduce aggression (Kazdin, 2008) – may be most effective when structured around the pattern of horizontal and vertical individualism and collectivism within which the adolescent resides. For instance, if an adolescent with aggression problems has high individualism in an individualistic society (e.g., the U.S.), increasing their awareness of the reality of negative consequences of aggression for themselves (e.g., being suspended at school; arrest) may be most useful. However, in a country like Vietnam, for an adolescent with high levels of vertical collectivism, focusing on awareness of the potential status consequences of aggression for their family, such as negative reactions of individuals higher in the hierarchy, may be more useful. This would also apply to reinforcement of desired alternative behavior (i.e., non-aggressive behavior), which is seen is as or more important than punishment for reducing aggression (Kazdin, 2008). In the present case of vertical collectivism this might consist of, for instance, helping the adolescent understand that being a role model for siblings or peers of non-aggressive but adaptive responses to ACE (e.g., sharing with a trusted adult) would be a high-status way to serve the family and community. This also could also serve to reduce anger (a cause of reactive aggression) at being mistreated, by being respected within the hierarchy. Further, as the multi-component structure of the Social Control Theory emphasizes, other culturally-appropriate components for such interventions would be critical. For instance, providing training in emotion regulation and other standard evidence-based coping skills would be important so that the adolescent has the skills, as well as the motivation, to choose courses of action other than aggression that address the factors underlying their aggression.

### Study Limitations and Future Directions

The study has several limitations important to consider in interpreting its findings but that suggest potentially useful directions for future research. The study used a cross-sectional design and it thus was not possible to assess direction of causality. Although analyses were structured with ACEs as the cause / independent variables, and adolescent aggression as the outcome / dependent variables, the other direction of causality is possible with, for instance, an adolescent’s aggression resulting in parental anger and consequent abuse of the child. Third variable explanations, such as a family’s low economic status causing stress resulting in both parental child abuse and adolescent aggression also cannot be ruled out. Thus, longitudinal research will be important. However, longitudinal research focused on ACEs is complex, as ACEs tend to be repeated over an extended time span, up to 18 years (Xiao et al., 2024). Consider, for instance, an adolescent assessed at age 15 (T1) and then one year (T2) later. If this adolescent reported at T1 that he or she had been struck by a parent with an object multiple times over the past year (age 14 to 15), but the longitudinal relation between T1 ACEs and T2 adolescent aggression (controlling for T1 adolescent aggression) was non-significant, it is possible that this reflects a lack of causal effect. But it is also possible that the parent has been striking the child with an object for a number of years, and that effects of this child abuse have become stable, and hence do not predict change in adolescent aggression from T1 to T2 in the study.

Also, assessment of moderator effects of collectivism and individualism involving other mental health problems, in particular emotional mental health problems, will be important. This study focused on adolescent aggression, but ACEs are linked to other mental health problems such as depression and anxiety (Strathearn et al., 2020b). Because emotional problems are more self-focused than behavioral conduct problems (Butcher & Kendall, 2018), it is possible that vertical collectivism might be a protective factor for aggressive behavior – directed towards others – but a risk factor for emotional problems such as depression – directed towards the self. This would have important intervention implications.

## Conclusions

A central implication for future research is the importance of the horizontal and vertical sub-dimensions of collectivism and individualism for understanding how these cultural constructs impact mental health. The moderator effect size of vertical collectivism on proactive aggression was significantly (and 3.5 times) larger than the second largest moderator effect size, horizontal collectivism on proactive aggression. This highlights these dimensions’ utility for more precisely identifying potential mechanisms, in that it may be awareness and acceptance of the reality of the power hierarchy rather than simply a focus and concern for one’s groups that underlie moderator effects seen in this (and potentially other) studies.

Finally, it has been found that as societies develop economically, levels of individualism tend to increase and levels of collectivism decrease (Triandis & Gelfand, 2012). One consequence of this may be that adaptive features of collectivism at a societal level, in the present case reduction ACEs potential effects on adolescent aggression, may be lost. Understanding the processes underlying present study results potentially could provide guidelines for societies as cultural changes occur. Reducing or eliminating ACEs would of course be optimal, but given the ubiquity and persistence of ACEs across time and cultures (World Health Organization, 2020), it will be important to develop supportive guidelines (e.g., how to maintain specific adaptive aspects of collectivism) for if and when such protective factors decrease.

## Electronic Supplementary Material

Below is the link to the electronic supplementary material.

Supplementary Material 1

## Data Availability

The data used for this study are available for study replication purposes upon reasonable request to Bahr Weiss, at bahr.weiss@vanderbilt.edu.
