# Supplementary material for: Individualism and Collectivism as Moderators of Relations between Adverse Childhood Experiences and Adolescent Aggressive Behavior
Source: Res Child Adolesc Psychopathol. 2025 Feb 27;53(4):569–81. doi: 10.1007/s10802-025-01296-z (PMC12031781; doi:10.1007/s10802-025-01296-z)
Supplement: Supplementary file 1 — Supplementary Material 1 [file 10802_2025_1296_MOESM1_ESM.docx]

**Supplemental Materials #1 – Construct Validity of the INDCOL Scale / Table S1**

A series of analyses were conducted to evaluate the construct validity of the INDCOL measure within our sample of Vietnamese adolescents. The analyses were structured around previous research that has identified two key aspects of Vietnamese society, that would be linked to the validity of the questionnaire. First, collectivism (vs. individualism) has been found to be a defining feature of Vietnamese society (Kelley & Sasges, 2024); in Hofstede’s international ratings of individualism vs. collectivism at the national level, Vietnam is rated as one of the most highly collectivistic countries (Hofstede, 2024). Second, research has identified Vietnamese society as being strongly hierarchical with a pronounced focus on hierarchical relationships and power-distance structure (London, 2022), although this characteristic is seen as somewhat less central than collectivism (Hofstede, 2024).

Based on these research findings, we made the following predictions regarding the INDCOL measure that, if confirmed, would support its construct validity:

(1) We predicted that in a repeated measures analysis assessing levels of the four subscales, structured as a 2 (Individualism vs. Collectivism) by 2 (Horizontal vs. Vertical) analysis, there would be a significant effect for Individualism vs. Collectivism, with the levels of collectivism significantly higher than levels of individualism. This would support the validity of the measure, given such as result’s congruence with the literature that Vietnam society is strongly collectivistic.

(2) Second, we predicted that in the repeated measures analysis, there would also be a significant effect for Horizontal vs. Vertical, with the levels of Vertical structure (focus on hierarchical relationships) significantly higher than levels of Horizontal structures (focus on egalitarian relationships). If this prediction were confirmed, this would support the measure’s construct validity, given such result’s congruence with literature indicating that Vietnam is a hierarchically focused and structured society.

(3) Third, we predicted that in the repeated measures analysis, there would be a significant interaction effect for Individualism/Collectivism (IV) X Horizontal/Vertical (HV), with the effect of HV larger for collectivism than for individualism. That is, we predicted that the CV - CH contrast would be larger than the IV – IH. This prediction was made based on the literature cited above indicating that vertical structures are important in Vietnamese society, but that the most defining feature of Vietnamese society is collectivism. Hence, it was predicted that the difference between vertical collectivism and horizontal collectivism would be larger than the difference between vertical individualism and horizontal individualism.

To test these predictions, a repeated measures analysis was conducted in SAS (9.4), with the four INDCOL subscales structured into a (Inv/Col) + (Hor/Ver) + (Inv/Col X Hor/Ver) repeated measures model. All three of the predictions were confirmed, supporting the construct validity of the measure. For the first prediction, the effect of CI (Collectivism vs. Individualism) was significant, F(1,643) = 315.16, p <.0001, R^2^ = 0.33. As Supplemental Table S1 below reports, levels of Collectivism were significantly higher than levels of Individualism (with an effect size of R^2^ = 0.33). For the second prediction, the effect of HV (Horizontal vs. Vertical) was significant, F(1,643) = 78.58, p <.0001, R^2^ = 0.11, with levels of vertical values significantly higher than levels of horizontal values, R^2^ = 0.11. The fact that the R^2^ for CI was 3 times the R^2^ for HV also supports that validity of the INDCOL, given collectivism being seen as more central to Vietnamese society than vertical structures. For the third prediction, the interaction between these two factors was significant, F(1,643) = 46.35, p <.0001, R^2^ = 0.07. As Supplemental Table S1 shows, the effect of HV (Horizontal vs. Vertical) was significant for collectivism but not for individualism, supporting the predominance of collectivism as the key cultural factor.

| **Supplemental Table S1** | | | |
| --- | --- | --- | --- |
| **Mean levels (and SD) of INDCOL subscales** | | | |
|  | **Horizontal** | **Vertical** | **IC** |
| **Collectivism** | 6.43 (1.33) ^A, X^ | 7.12 (1.42) ^B, X^ | 6.78 (1.15) ^X^ |
| **Individualism** | 5.68 (1.35) ^A, Y^ | 5.77 (1.53) ^A, Y^ | 5.73 (1.18) ^Y^ |
| **HV** | 6.06 (1.00) ^A^ | 6.45 (1.11) ^B^ |  |
| Notes: Columns with the same A or B superscript do not differ significantly across the row. Rows with the same X or Y superscript do not differ significantly across the column. | | | |

| **Supplemental Materials #2: Table S2 – Moderator effects of ACE Domain, on IC/HV interaction effects** | | | | |  |
| --- | --- | --- | --- | --- | --- |
| Dependent Variable | IC/HV Moderator | F (7, 587) | ω^2^ |  |  |
| Proactive Aggression | Horizontal Collectivism | 1.11 |  |  |  |
|  | Vertical Collectivism | 2.50* | 0.03 |  |  |
|  | Horizontal Individualism | 1.62 |  |  |  |
|  | Vertical Individualism | 1.51 |  |  |  |
| Reactive Aggression | Horizontal Collectivism | 1.94 |  |  |  |
|  | Vertical Collectivism | 1.31 |  |  |  |
|  | Horizontal Individualism | 1.39 |  |  |  |
|  | Vertical Individualism | 0.75 |  |  |  |
| Notes. Effect of interest in these analyses is the ACE Domain x IC/HV interaction, with all underlying main and interaction effects included in the model. | | | | | |

| **Supplemental Materials #3: Table S3 – Moderator effects of Location, on ACE x IC/HV interaction effects** | | | | |  |
| --- | --- | --- | --- | --- | --- |
| Dependent Variable | IC/HV Moderator | F (1, 636) | ω^2^ |  |  |
| Proactive Aggression | Horizontal Collectivism | 9.85** | 0.02 |  |  |
|  | Vertical Collectivism | 0.06 |  |  |  |
|  | Horizontal Individualism | 0.73 |  |  |  |
|  | Vertical Individualism | 1.80 |  |  |  |
| Reactive Aggression | Horizontal Collectivism | 0.44 |  |  |  |
|  | Vertical Collectivism | 2.72 |  |  |  |
|  | Horizontal Individualism | 1.21 |  |  |  |
|  | Vertical Individualism | 0.87 |  |  |  |
| Notes. Effect of interest in these analyses is the ACE x IC/HV x Location interaction, with all underlying main and interaction effects included in the model. | | | | | |

**Supplemental Materials #4: Figures**


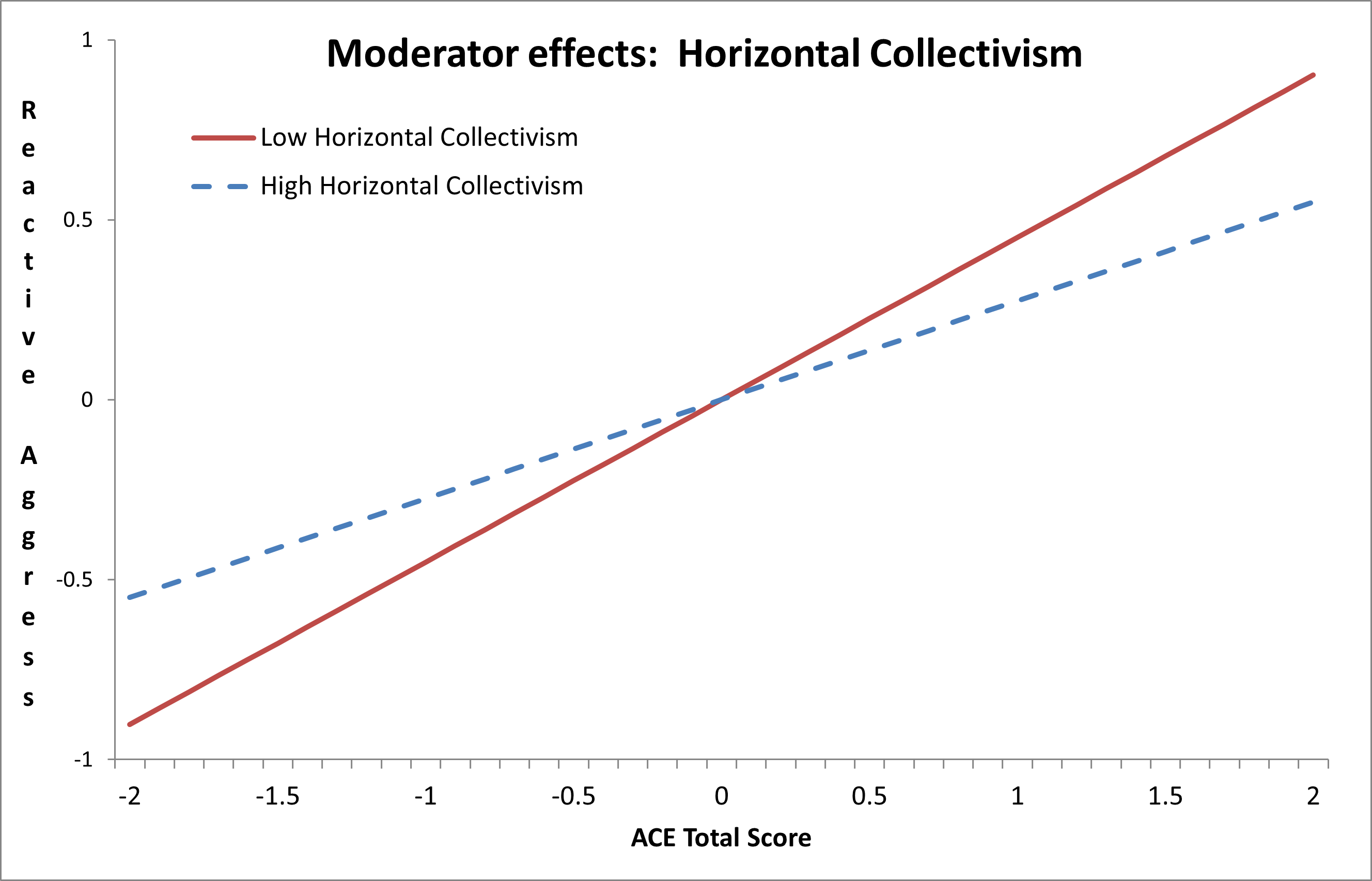

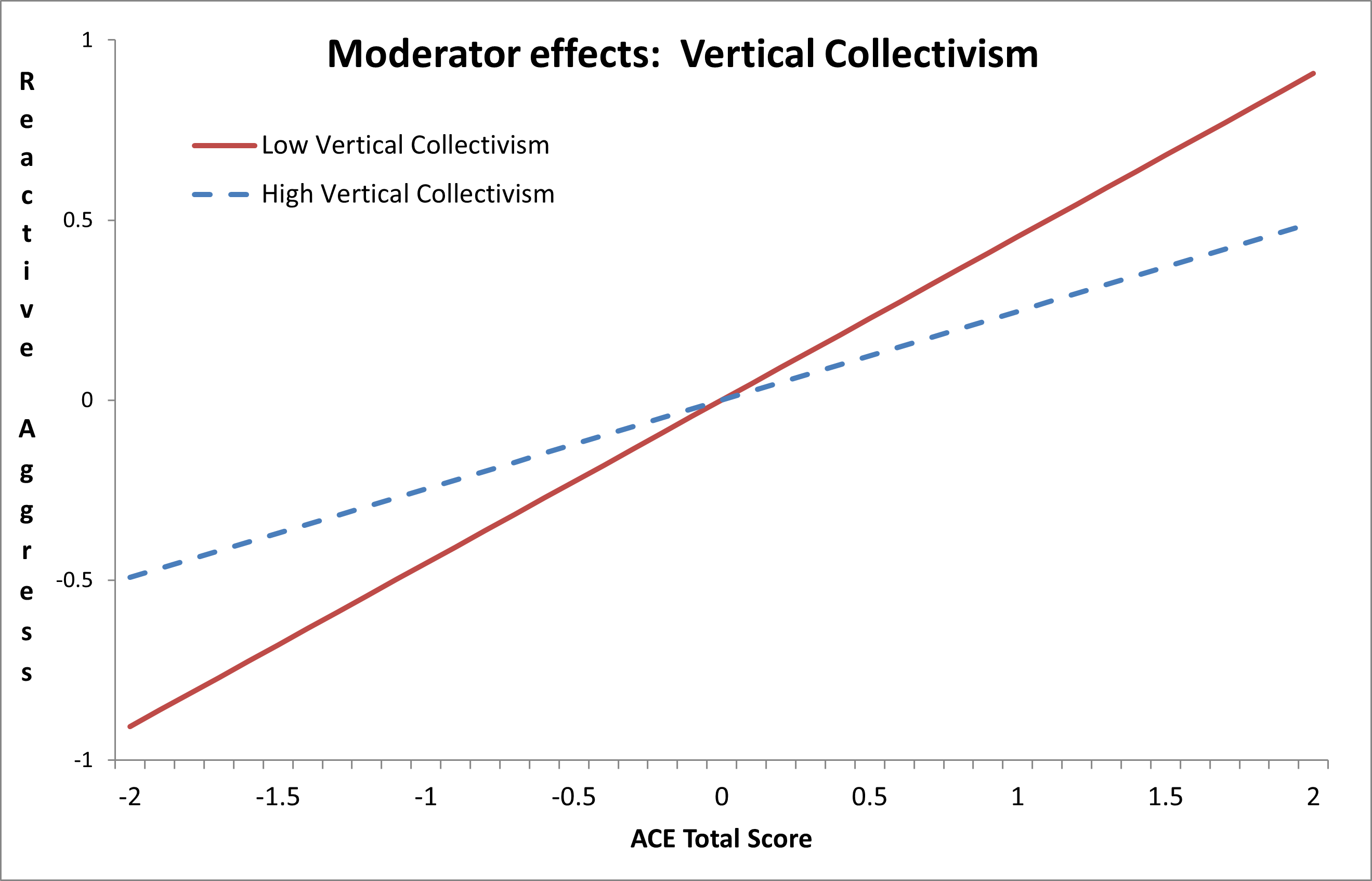

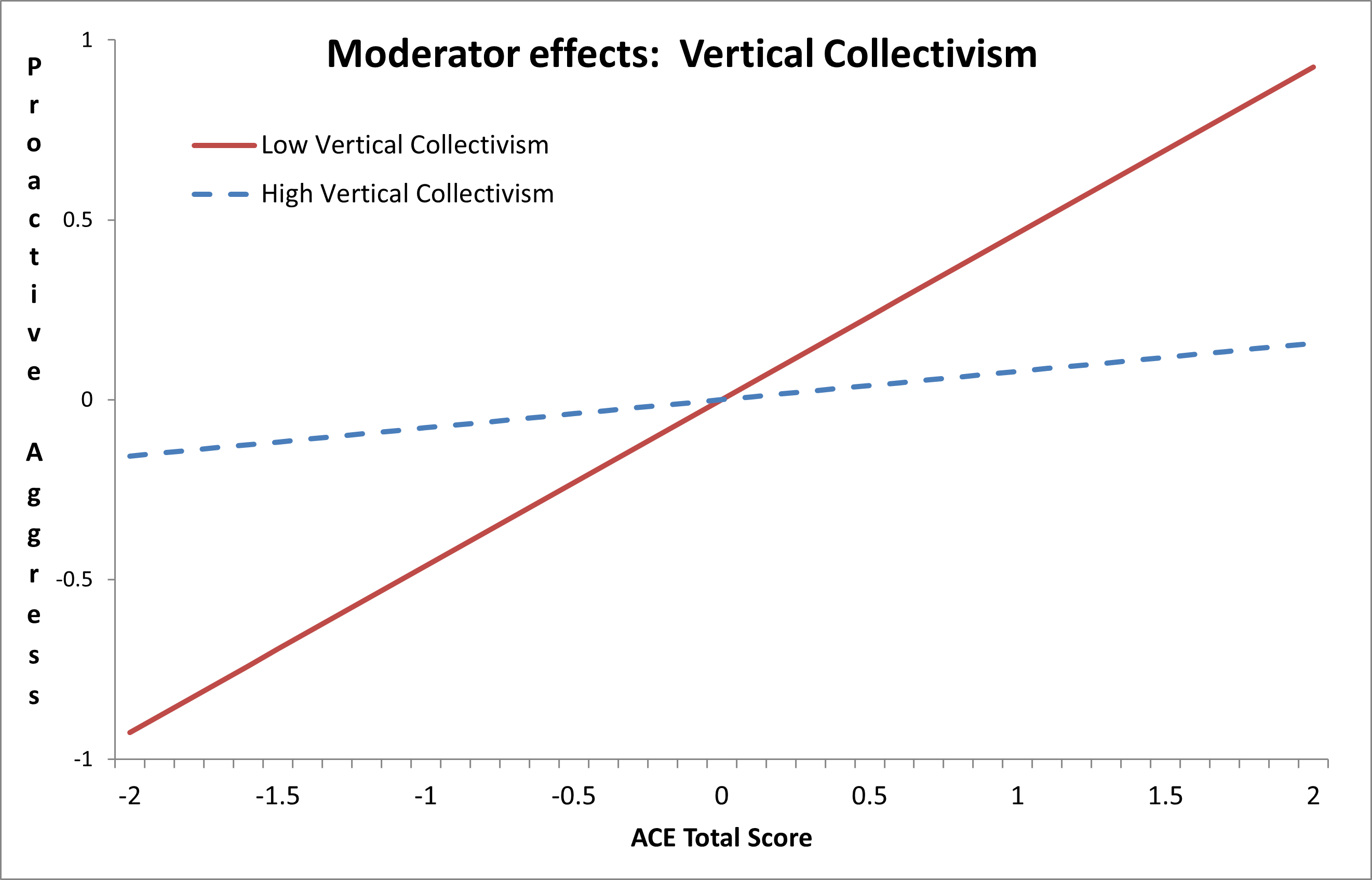

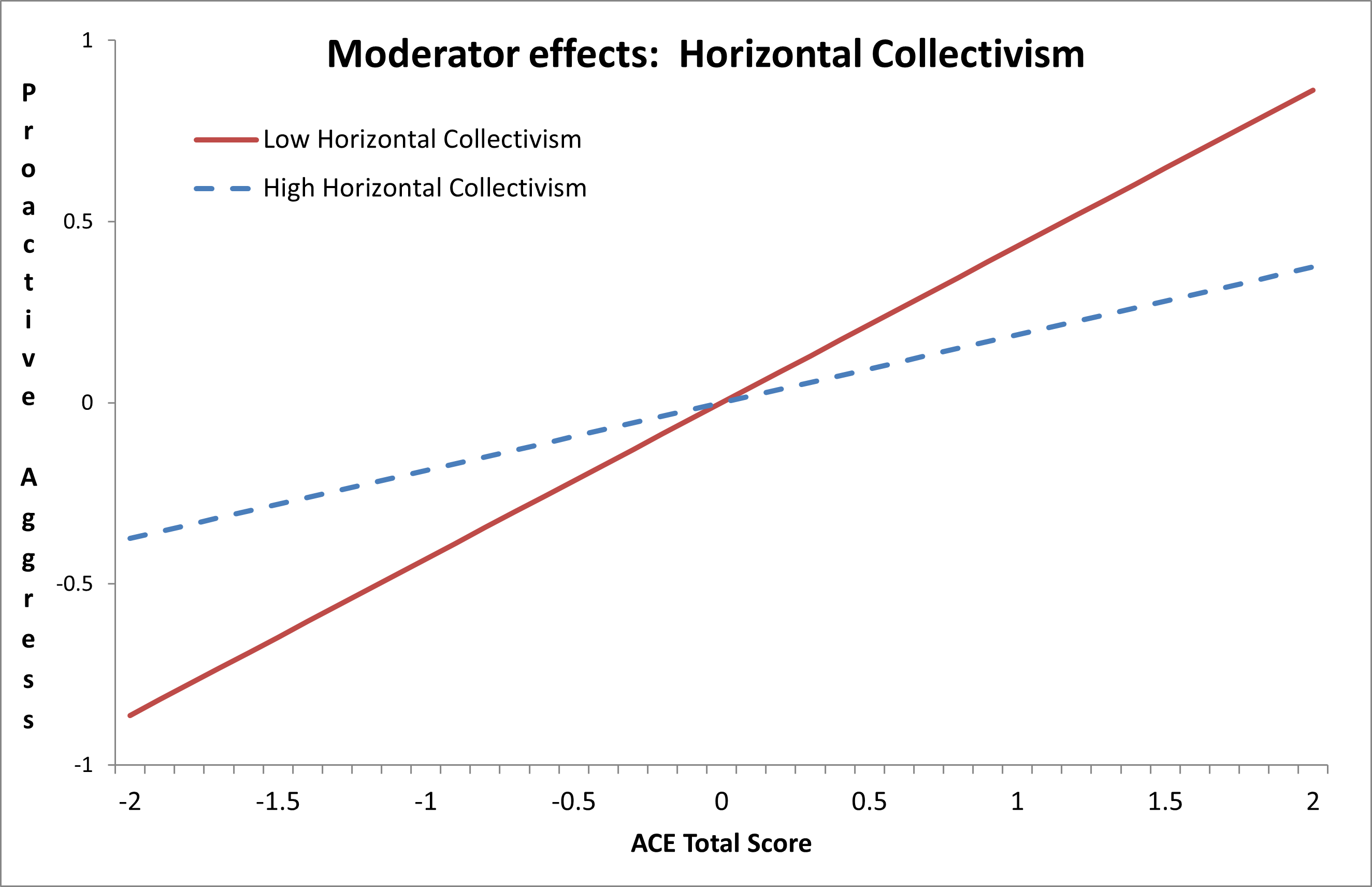


Figure 1. Moderator effects of Collectivism on relations between ACE and Aggression. X and Y axes represent standardized deviations from the mean of the variable. All four of the moderator effects are significant.


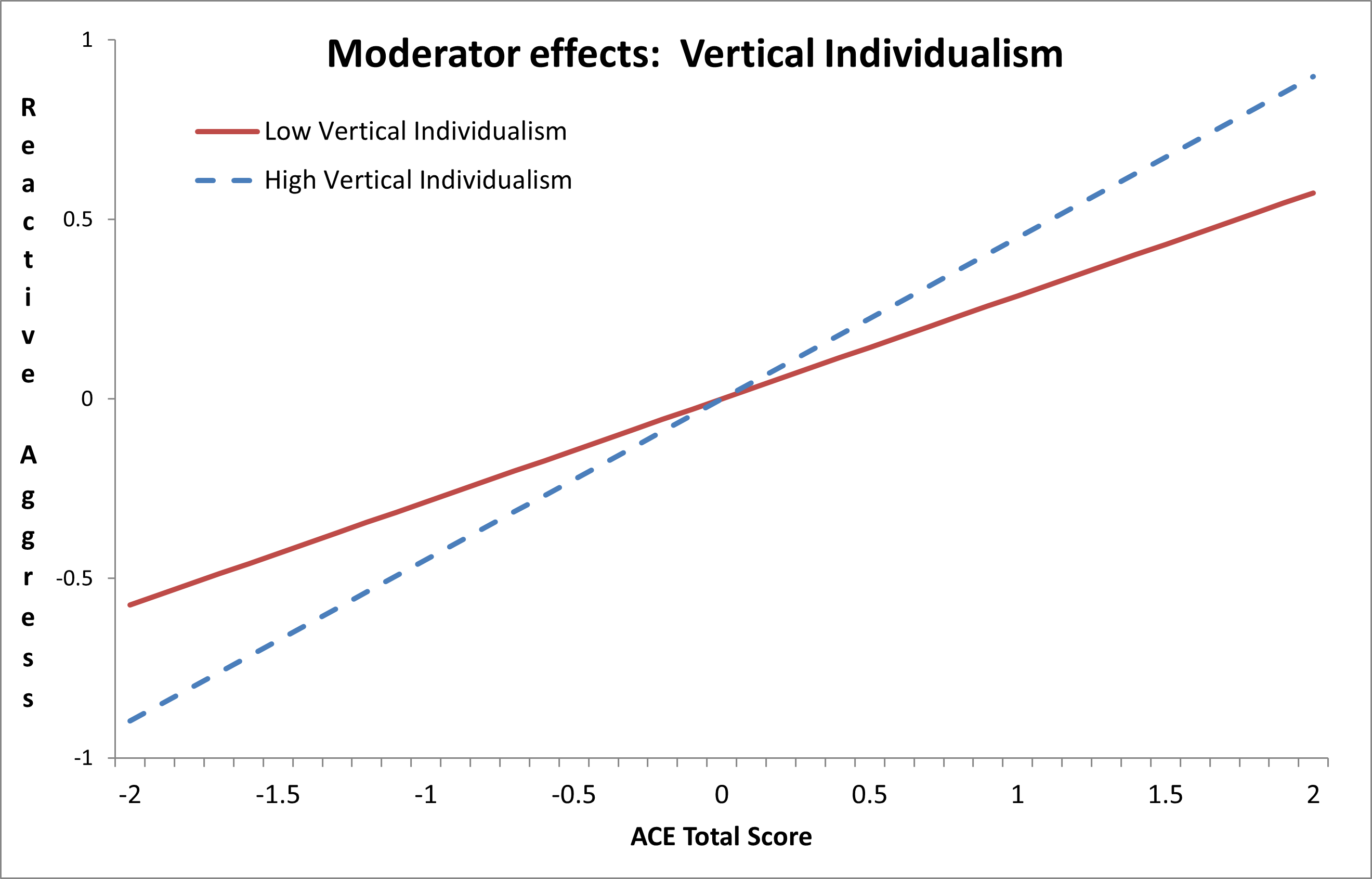

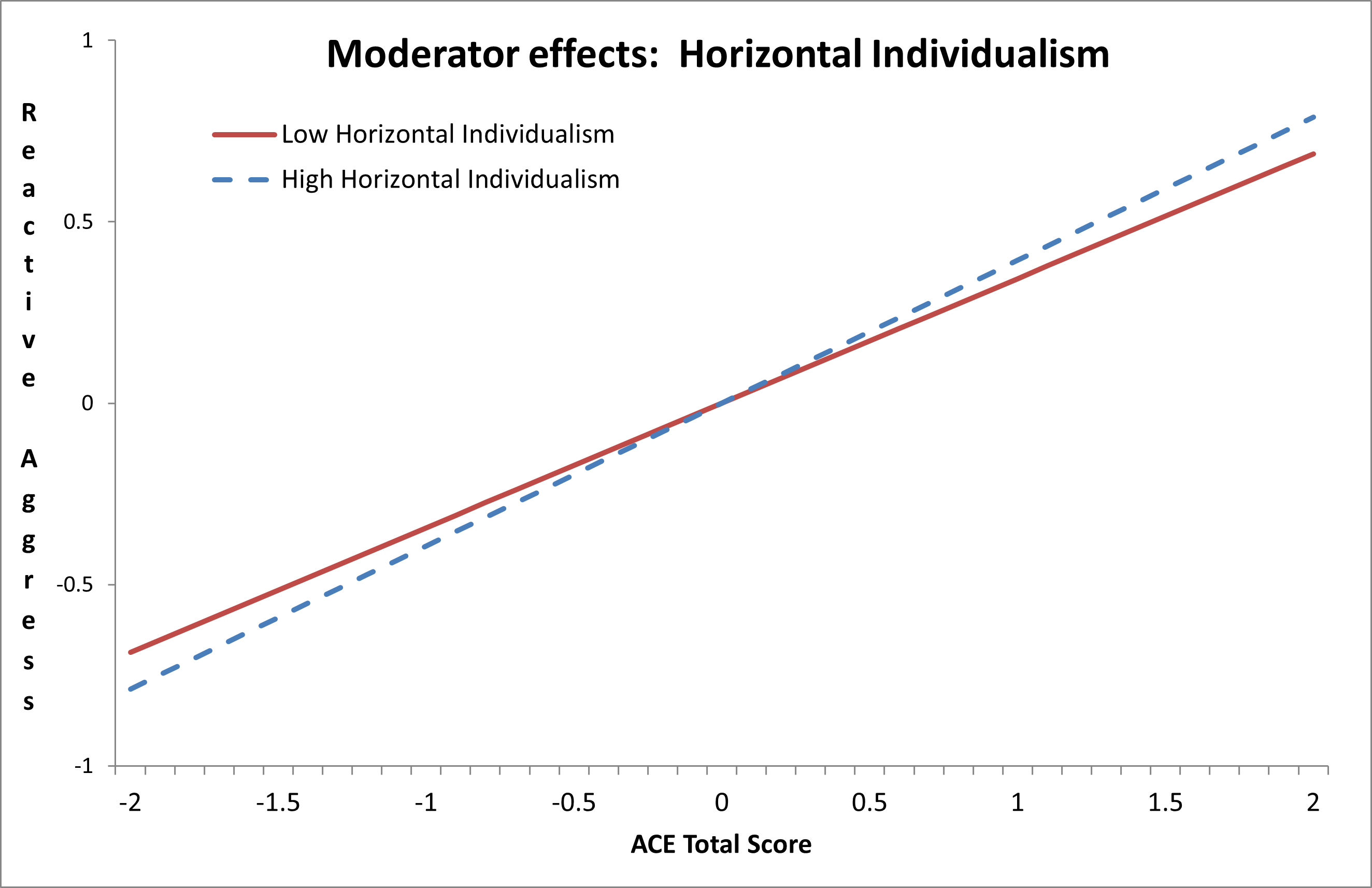

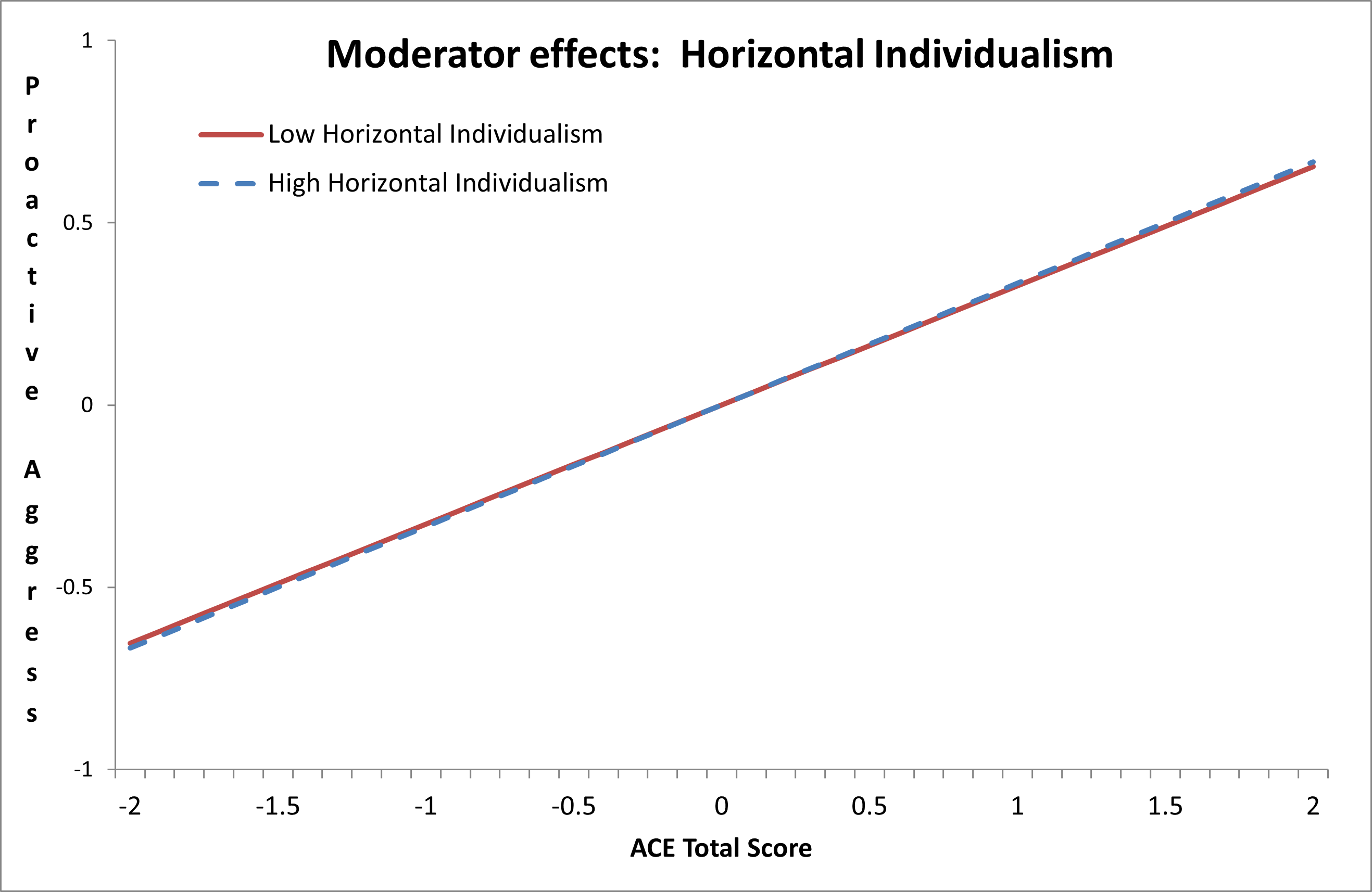


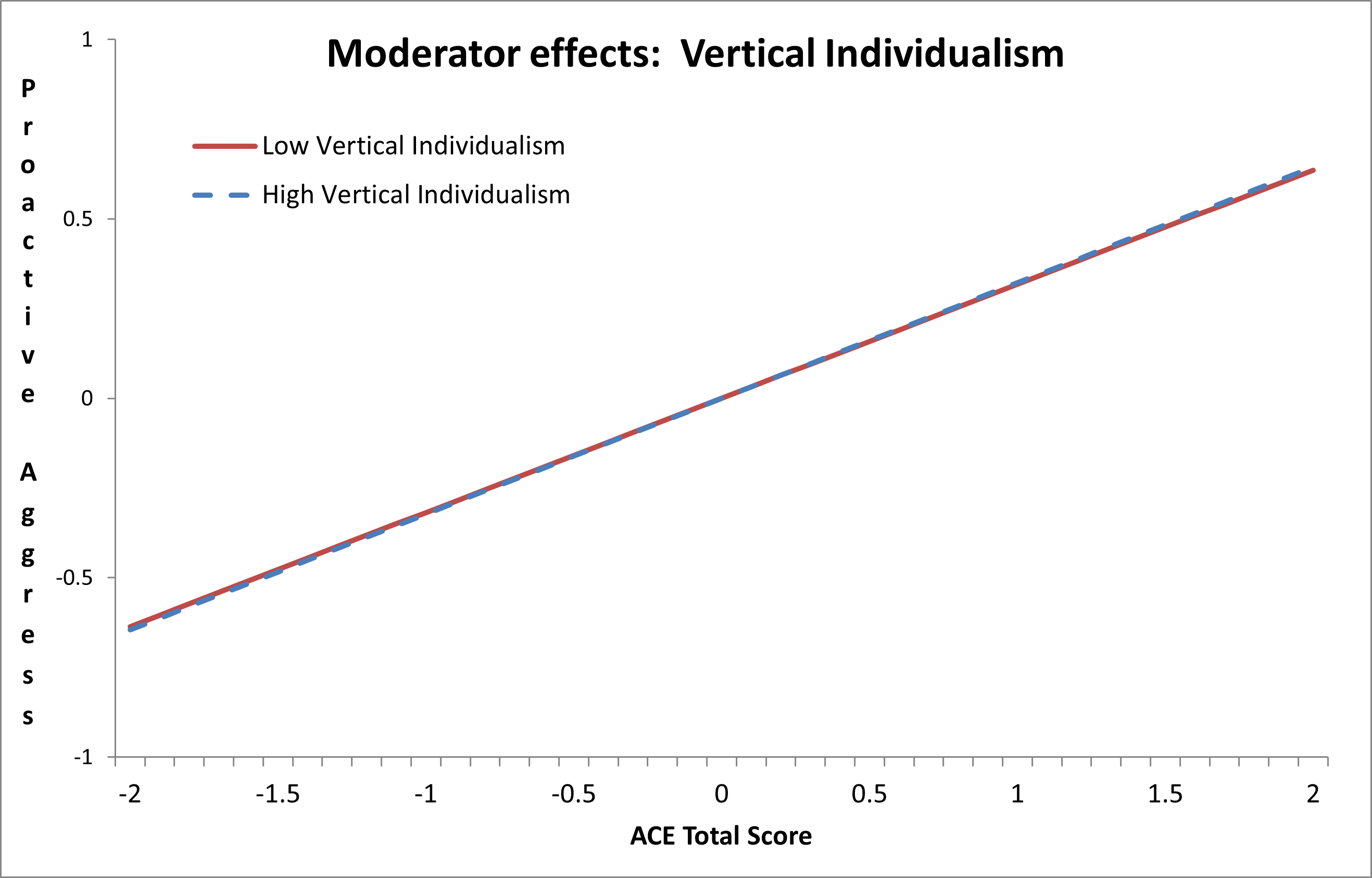


Figure 2. Moderator effects of Individualism on relations between ACE and Aggression. X and Y axes represent standardized deviations from the mean of the variable. Only the Vertical Individualism moderator effect on Reactive Aggression was significant.
